# Cell cycle phase perturbations and apoptosis in tumour cells induced by aplidine

**DOI:** 10.1038/sj.bjc.6600265

**Published:** 2002-05-06

**Authors:** E Erba, L Bassano, G Di Liberti, I Muradore, G Chiorino, P Ubezio, S Vignati, A Codegoni, M A Desiderio, G Faircloth, J Jimeno, M D'Incalci

**Affiliations:** Cancer Pharmacology Laboratory, Department of Oncology, Istituto di Ricerche Farmacologiche “Mario Negri”, via Eritrea 62, 20157 Milan, Italy; Institute of General Pathology and CNR Center for Research on Cell Pathology, University of Milan, via L Mangiagalli 31, 20133 Milan, Italy; PharmaMar USA, Inc., 320 Putnam Avenue, Cambridge, Massachusetts, MA 02139, USA; PharmaMar SA, Research and Development, Calle de la Calera 3, 28760 Tres Cantos, Madrid, Spain

**Keywords:** natural compound, Aplidine, cell cycle, apoptosis

## Abstract

Aplidine, dehydrodidemnin B, is a marine depsipeptide isolated from the Mediterranean tunicate *Aplidium albicans* currently in phase II clinical trial. In human Molt-4 leukaemia cells Aplidine was found to be cytotoxic at nanomolar concentrations and to induce both a G_1_ arrest and a G_2_ blockade. The drug-induced cell cycle perturbations and subsequent cell death do not appear to be related to macromolecular synthesis (protein, RNA, DNA) since the effects occur at concentrations (e.g. 10 nM) in which macromolecule synthesis was not markedly affected. Ten nM Aplidine for 1 h inhibited ornithine decarboxylase activity, with a subsequently strong decrease in putrescine levels. This finding has questionable relevance since addition of putrescine did not significantly reduce the cell cycle perturbations or the cytotoxicity of Aplidine. The cell cycle perturbations caused by Aplidine were also not due to an effect on the cyclin-dependent kinases. Although the mechanism of action of Aplidine is still unclear, the cell cycle phase perturbations and the rapid induction of apoptosis in Molt-4 cells appear to be due to a mechanism different from that of known anticancer drugs.

*British Journal of Cancer* (2002) **86**, 1510–1517. DOI: 10.1038/sj/bjc/6600265 www.bjcancer.com

© 2002 Cancer Research UK

## 

Aplidine, dehydrodidemnin B (Figure 1

**Figure 1:**
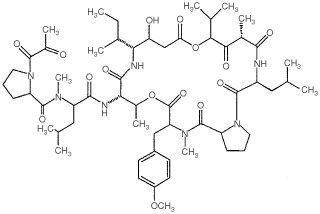
Aplidine chemical structure.

, chemical structure), is a marine depsipeptide isolated from the Mediterranean tunicate *Aplidium albicans*, that is structurally related to didemnin B, and has been shown to be *in vitro* active against both human haemathological and solid tumour cell lines (Urdiales *et al*, 1996; Lobo *et al*, 1997; Depenbrock *et al*, 1998; Geldof *et al*, 1999), Moreover, Aplidine has been shown to be active against *in vivo* murine B16 melanoma and in several human tumours growing in athymic mice (Faircloth *et al*, 1995, 1996).

It has been described that didemnins inhibit the progression from G_1_ to S phase of the cell cycle (Crampton *et al*, 1984). Several biochemical effects of didemnins have also been reported: inhibition of protein synthesis via GTP-dependent elongation factor 1-alpha *in vitro* (Crews *et al*, 1994), and of protein, DNA and RNA syntheses in different cell lines (Crampton *et al*, 1984; Sakai *et al*, 1996) Inhibition of the ornithine decarboxylase (ODC) activity by didemnins was previously described (Urdiales *et al*, 1996), but the relevance of this biochemical effect for the antitumour activity of Aplidine has not been shown.

The present study purpose is to better characterise the mechanisms involved in the Aplidine-induced antiproliferative effect, cell cycle perturbations and cell death induced in human Molt-4 leukaemia cell line.

## MATERIALS AND METHODS

### Drug treatment

Aplidine (Figure 1) was generously provided by PharmaMar. The cytotoxic effect of Aplidine on Molt-4 cells was evaluated by exposing the cells for different times to the drug and by counting the number of cells at different time intervals after drug-washout by a Coulter Counter instrument.

### Cell cycle

#### Monoparametric DNA analysis and cell flux simulation

Exponentially growing Molt-4 cells were treated for 1 h with 0, 1, 5, 10, 20 or 30 nM Aplidine. At 24, 48 and 72 h after drug-washout the number of cells was evaluated by counting the cells with a Coulter Counter instrument, and the cells were fixed in 70% ethanol and kept at 4°C before DNA staining with propidium iodide. DNA flow cytometric analysis were performed on at least 10 000 cells using a FACSCalibur system (Becton Dickinson, Sunnyvale, CA, USA). The coefficient of variation of the G_1_ peak of the Molt-4 fixed cells was between 2 and 2.5%. The DNA histograms of each sample were analysed and the percentages of cells in each cell cycle phase were derived by means of a home-made fitting programme based on the sum-of-gaussians method (Ubezio, 1985; Sena *et al*, 1999). However the direct analysis of the experimental data expressed as percentage of cells in a given phase can be misleading if there is a concomitant cell cycle block with killing in one or different phases of the cell cycle. In fact if the drug kills in one phase and blocks in the same phase the percentage of cells in that phase might be similar to that of untreated control cells. This is explained by the fact that a cell killing effect results in a decrease of the percentage and the block is a relative increase in that percentage. The relative importance of the two effects, i.e. blocking and cell killing can vary overtime making the interpretation of the data virtually impossible. In addition when the block is overcome there might be a wave of synchronisation with changes in the relative distribution of cells in the different phases compared to asynchronous cell cycle distribution of control cells.

In order to overcome these interpretation problems we have developed and used a simulation programme, previously described in detail (Montalenti *et al*, 1998; Sena *et al*, 1999), based on the kinetic properties of the cell line.

The simulation programme forecasts the cell cycle flux in a cell population, starting from the cell cycle distribution at a given time hypothesising the probability that a cell is blocked or killed in a cell cycle phase. The time-course of cell counts and of %G_1_, %S and %G_2_M are given as output and can be compared with actual data.

Thus, the programme enables us to test a hypothesis on the time-course of blocking and killing that can occur in each phase to correlate with the data. The latest version of the programme runs on a personal computer using Microsoft Excel and is available from the author (ubezio@marionegri.it).

#### Biparametric BrdUrd/DNA analysis

During the last 15 min of 1 h Aplidine exposure, 20 μM bromodeoxyuridine (BrdUrd) was added to the cells. After treatment the cells were washed twice with PBS and fresh medium was provided. After 1 h treatment and at 4, 8, 12, 24, 29, 36 and 48 h after drug-washout control and treated cells were fixed in 70% ethanol and kept at 4°C before BrdUrd/DNA flow cytometric analysis (Erba *et al*, 2001). With this protocol it was also possible to obtain a distinct evaluation of cell cycle perturbations in cells which were in S phase (BrdUrd positive cells) or in G_1_ or in G_1_M phases (BrdUrd negative cells) during 1 h 10 nM Aplidine exposure.

### Ornithine decarboxylase (ODC) activity assay

Frozen cells (5×10^6^) were sonicated in 100 μl of cold Tris-HCl (pH 7.2) containing 0.1 mM EDTA and 2 mM DTT and were subsequently centrifuged at 14 000 r.p.m. for 20 min in an Eppendorf microcentrifuge at 4°C. ODC activity was measured in the supernatants as release of labelled CO_2_ from [1-^14^C]ornithine (56 Ci mol^−1^, Amersham Pharmacia Biotech, Milan, Italy), as previously reported (Desiderio *et al*, 1993). The reaction mixture (final volume 250 μl) contained cytosol supernatant (about 150 μg of protein), 50 mM Tris-HCl (pH 7.1), and 1 mM [1-^14^C]ornithine (sp. act. 4 Ci mol^−1^). Blanks were prepared in the presence of 4 mM α-difluoromethylornithine (generously given by Marion Merrell Dow Research Institute, Strasbourg, France) to inhibit ODC activity. ODC activity is reported as pmol of CO_2_ formed in 1 h per mg of protein. Protein content in cytosol extracts was assayed by the standard method (Lowry *et al*, 1951).

### Polyamine determination

Frozen cell samples (3×10^6^) were sonicated in 80 μl of 0.2 N PCA and then centrifuged at 5000 r.p.m. in an Eppendorf microcentrifuge for 20 min. Aliquots of supernatants were analysed by HPLC (Perkin-Elmer Corp., Norwalk, CN, USA) using a C_18_ reverse-phase column (4 μm particle size, 150×3.9 mm, Waters). Evaluation of polyamines was performed after post-column derivatisation with *o*-phtaldialdehyde and fluorescence detection (Desiderio, 1992).

### DNA, RNA and proteins synthesis assay

Exponentially growing cells were treated with different concentrations of Aplidine. At different drug exposure time intervals 1 μCi ml^−1^ [^3^H]thymidine, or 1 μCi ml^−1^ [^3^H]uridine, or 2 μCi ml^−1^ [^3^H]leucine were added to the cells for 2 h and the radioactivity was evaluated by using a liquid scintillator counter.

### Extracts preparation, Western blotting analysis and CDK activity

Cell extracts were prepared by addition of lysis buffer containing protease inhibitors according to standard procedures (Sambrook *et al*, 1989). Extracts corresponding to 100 mg of protein were separated on 12% SDS–PAGE, blotted onto nitrocellulose membranes, and incubated with antibodies against cyclins B, D1 and E, Cdk2, Cdk4, Cdc2, p21, p16, p53, pRb, Bax and Caspase 3 (Santa Cruz Biotech.,Inc., Heidelberg, Germany). As secondary antibodies, horseradish peroxidase labelled anti-rabbit or anti-mouse antibodies (Santa Cruz Biotech) were used. Protein bands were visualised by enhanced chemiluminescence. Kinase activity was measured as previously reported (Bonfanti *et al*, 1997)

### Detection of apoptotic process

The fraction of apoptotic cells after Aplidine exposure was evaluated by Terminal-dUTP-Transferase (TdT) flow cytometric assay as previously described (Bergamaschi *et al*, 1999).

## RESULTS

### Growth inhibition

**Figure 2:**
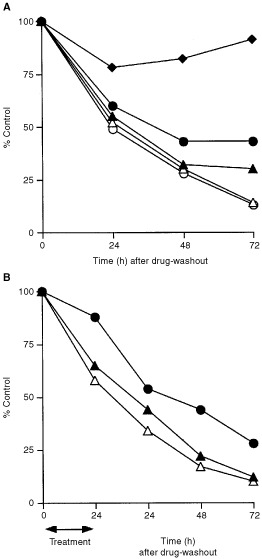
Effect of 1 h (**A**) or 24 h (**B**) Aplidine exposure on cell growth evaluated at different time intervals after treatment and drug-washout. Each point is the mean of three replicates; bars represent the standard deviation. Solid diamond=Aplidine 1 nM; solid circle=Aplidine 5 nM; solid triangle= Aplidine 10 nM; open triangle=Aplidine 20 nM; open circle=Aplidine 30 nM.

Aplidine was active at nanomolar concentrations, which induced a dose-dependent growth inhibition in Molt-4 cells (Figure 2

) and the effects appeared to be similar at 1 h (A) and 24 h treatment time (B).

### Cell cycle studies

**Figure 3:**
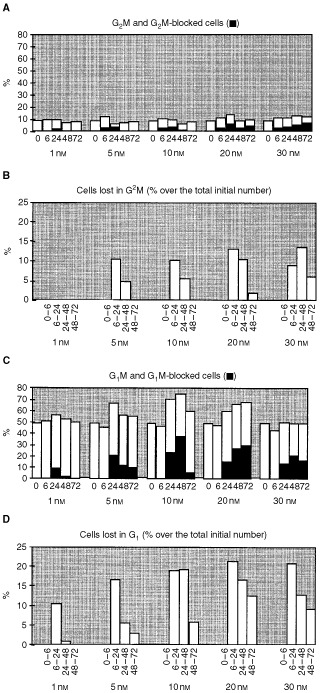
(**A**–**C**) Percentages of cells in G_2_M (**A**) and G_1_ (**C**) cell cycle phases. The black columns indicate the percentage of blocked cells in G_2_M or G_1_, as resulting from simulation of experimental cell cycle percentages and absolute cell counts. (**B**–**D**) Percentage of cell cohort, from the overall initial population, which have died during G_2_M (**B**) or G_1_ (**D**) block and evaluated at different time intervals after drug-washout (simulation results).

We were interested in evaluating the drug's effects on G_1_, S and G_2_M phases separately to distinguish blocking or delaying effects from killing ones. Figure 3

shows the experimental percentages of G_1_ and G_2_M at different times after Aplidine treatment, compared to the amount of blocked or lost cells resulting from the simulation. The computer simulation allowed us to discard any data not including all the effects of block and killing in each phase reported below. The chosen simulation reproduces experimental cell cycle percentages within 2% and experimental cell counts within 10%. Notice that the method allows us to estimate the percentage of cells blocked in G_1_ and G_2_M and the number of dead cells, distinguishing between cells that have died when they were blocked in G_1_ or in G_2_M.

As shown in Figure 3A, the simulation required the introduction of a partial G_2_M block to reproduce the experimental data at concentrations as low as 5 nM. Among the cells exiting from G_2_M between 0 and 6 h (2.6% of the whole cell population per hour in controls), the probability of being blocked ranged from 15% (5 nM) to 22% (30 nM) resulting in the 2.3–3.2% cells in the G_2_M block at 6 h. According to the simulation, after 48 h drug-washout, G_2_M blocked cells were not detectable (<1%) using ⩽10 nM Aplidine, due to either their death or exit from the block, while at higher concentration blocked cells were detected up to 72 h despite a continuous loss (Figure 3A,B). Mortality in G_2_M peaked around 24 h with any Aplidine concentration from 5 to 30 nM.

A G_1_ block was suggested by the simulation, at any concentration starting from 24 h after drug-washout, even when the observed per cent G_1_ was not increasing over time after treatment with 30 nM Aplidine (Figure 3C,D). The percentage of G_1_ blocked cells after treatment with the concentrations higher than 10 nM increased from 24 to 48 h, indicating that even those cells that entered G_1_ between 24 and 48 h were susceptible to arrest. A long-lasting continuous G_1_ block, with 20 and 30 nM Aplidine, was suggested by the presence of G_1_ blocked cells at 72 h despite their loss. A different balance between arrest and death resulted in a different behaviour with lower concentrations: more blocked cells were obtained with 5 (9%) than with 10 nM (5%) at 72 h, due to the higher mortality with 10 nM. Although more cells died in G_1_ than in G_2_M, G_1_ lost cells were proportionally less than G_2_M lost cells after correction for the amount of cells present in these phases. For instance, with 20 nM, the number of cells that globally (in the 0–72 h interval) died in G_1_ was double than in G_2_M, but per cent G_1_ was on average four-fold per cent G_2_M and 29% cells were blocked in G_1_ against 4% in G_2_M at 72 h.

A temporary S phase delay was observed with intermediate doses; this effect became strong and persistent only at 30 nM Aplidine. No mortality effect was detected in S phase (data not shown).

Figure 4

**Figure 4:**
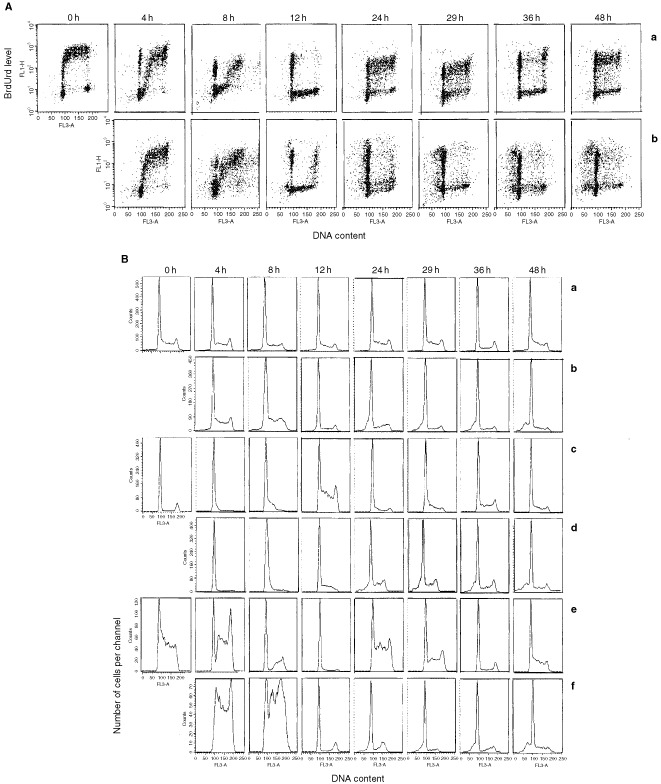
Effects of 1 h Aplidine exposure on the cell cycle phase distribution of Molt-4 cells evaluated at 0, 4, 8, 12, 24, 29, 36 and 48 h after drug-washout. During the last 15 min 10 nM Aplidine treatment, 20 μM BrdUrd was added to the cells, then the cells were washed with PBS and drug-free medium was provided. (**A**) Biparametric BrdUrd/DNA analysis of (a) control cells; and (b) Aplidine treated cells. (**B**) DNA histograms of (a) control cells (whole population); (b) Aplidine treated cells (whole population); (c) BrdUrd-negative control cells; (d) BrdUrd-negative Aplidine treated cells; (e) BrdUrd-positive control cells; and (f) BrdUrd-positive Aplidine treated cells. The data are representative of three independent replicates.

shows the effects on the cell cycle phase distributions caused by 1 h exposure with 10 nM Aplidine in Molt-4 cells evaluated by BrdUrd/DNA flow cytometric analysis at different time intervals after drug-washout. After Aplidine-washout, cells which were in the S phase (BrdUrd positive cells) during drug treatment, progressed through this phase of the cell cycle more slowly than control cells. At 8 h the fraction of cells that started a new cell cycle (G_1_ BrdUrd positive cell population) was higher in control (27%) rather than Aplidine treated cells (8%). At 24 h and up to 48 h after drug-washout the data are indicative of a G_1_ block, in agreement with simulation studies.

Aplidine was found to delay those cells that were in the G_1_ phase (BrdUrd negative cells) during drug treatment, from entering S phase. At 24 and up 48 h after Aplidine-washout the majority of the BrdUrd negative cells were blocked in the G_1_ phase too.

### ODC activity

**Figure 5:**
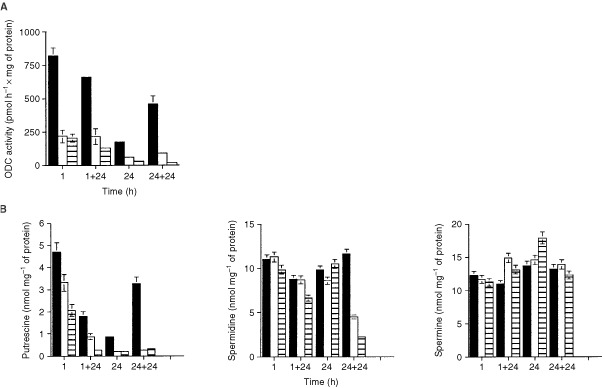
(**A**) Ornithine decarboxylase (ODC) activity evaluated after 1 and 24 h Aplidine treatment and at 24 h after drug-washout. The data are the mean±s.e. of three independent experiments performed in duplicate. 1=1 h Aplidine treatment; 1+24=1 h Aplidine treatment+24 h drug-washout; 24+24=24 h Aplidine treatment+24 h drug-washout; solid square=control cells; open square=10 nM Aplidine; striped square=20 nM Aplidine. (**B**) Putrescine, spermidine and spermine determinations performed after 1 and 24 h Aplidine treatment and at 24 h after drug-washout. The data are the mean±s.e. of three independent experiments performed in duplicate. 1=1 h treatment; 1+24=1 h treatment plus 24 h drug-washout; 24=24 h treatment; 24+24=24 h treatment plus 24 h drug-washout; solid square=control cells; open square=10 nM Aplidine; striped square=20 nM Aplidine.

As shown in Figure 5A

, 1 h exposure to 10 or 20 nM Aplidine caused an inhibition of ODC of 75%. The same percentage of inhibition was observed after 24 h of exposure. Aplidine *per se* did not affect ODC activity of a partially purified preparation, obtained by centrifugation of the cell homogenate at 14 000 r.p.m. for 20 min in an Eppendorf microcentrifuge at 20°C. This was shown by adding directly the drug (20 nM Aplidine) to test tubes containing all the components for ODC activity assay including the supernatant (about 150 μg of protein) (data not shown). At 24 h after drug-washout we observed a persistent inhibitory effect of the Aplidine on ODC activity. However, control ODC activity was stimulated by the addition of fresh medium (24+24).

In agreement with the decrease of ODC activity, we observed that putrescine levels diminished in Aplidine treated cells at 1 h (30 and 60% for Aplidine 10 and 20 nM respectively) and at 24 h (80% decrease) at both concentrations (Figure 5B). At 24 h after drug-washout (1 or 24 h treatment plus 24 h drug-washout) putrescine levels further decrease in Aplidine treated cells, while the control cells showed a huge increase probably due to fresh medium change that increased also ODC activity. Only spermidine level decreased (60–80%) at 24 h after drug-washout in 24 h Aplidine treated cells. At the other times of treatment higher polyamine (spermidine and spermine) levels were unaffected by Aplidine (Figure 5B).

To evaluate if decreased levels of putrescine could be important for the cytotoxic effect of Aplidine, 1 mM putrescine was added to the cells 2 h before 1 h Aplidine treatment. Then the drug-containing medium was removed and fresh medium containing 1 mM putrescine was added to the cells. Under these conditions the intracellular levels of putrescine were 6.44 nmol mg^−1^ protein in control cells, 16.8 nmol mg^−1^ protein putrescine pretreated control cells, 4.7 nmol mg^−1^ protein Aplidine treated cells and 16.9 nmol mg^−1^ protein in Aplidine treated cells preincubated with 1 mM putrescine. In spite of the fact that intracellular putrescine levels were comparable to that of control cells Aplidine was equally cytotoxic indicating that the cytotoxicity was not related to putrescine depletion (data not shown).

### Macromolecular synthesis

DNA, RNA and protein synthesis were evaluated after 1, 4, 8 and 24 h Aplidine exposure in Molt-4 cells. One hour Aplidine treatment at concentrations up to 50 nM did not cause a significant inhibition of DNA, RNA or protein synthesis. Inhibition of DNA, RNA and protein synthesis were obtained only at higher Aplidine concentrations (i.e. after 1 h with concentrations >100 nM) and/or a time exposures (i.e. after 8 h with concentrations ⩾50 nM; data not shown).

### Cell cycle related proteins

**Figure 6:**
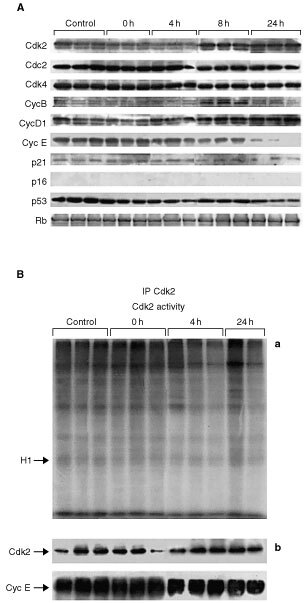
(**A**) Immunoblot analysis of Cdk2, Cdc2, Cdk4, cyclin B1, D1 and E, p21, p16, p53, and pRb expression in Molt-4 cells treated for 1 h with 10 nM Aplidine (three independent replicates). Molt-4 extracts were collected before and after treatment and, at 4, 8, 24 h after drug washout. One hundred μg of protein extracted from cells were immunoblotted with specific antibodies as described in Materials and methods. (**B**) (a) Histone H1 phosphorylation in Molt-4 cells lysates immunoprecipitated with Cdk2 antibodies. Cells were exposed for 1 h to 10 nM Aplidine (0 h) and then incubated in drug free medium (4 and 24 h after drug-washout) (three independent replicates). (b) Western blot analysis of Cdk2 and cyclin E in Cdk2 immunoprecipitates (three independent replicates).

We then evaluated the expression of proteins that regulate cell-cycle progression at the end of treatment with 10 nM Aplidine and 4, 8 and 24 h after drug-washout by Western blot analysis. As shown in Figure 6A

, the protein levels of Cdk2, Cdc2 and Cdk4, cyclin B and D1 remained constant at each time tested, with only a decline in cyclin E levels observable at 24 h after drug-washout. p16 levels were undetectable in Molt-4 lysates in agreement with the previously reported deletion of this gene in this cell line (Uchida *et al*, 1995).

The levels of p21, p53 and pRb, did not change under these experimental conditions.

Cdk2 Kinase activity measured at the end of 1 h treatment with 10 nM Aplidine (time 0 h) and at 4 and 24 h after drug-washout was not affected by drug treatment (Figure 6B). Cdk2 and cyclin E protein levels did not change in Western blot analysis of immunoprecipitates.

Similarly, Aplidine did not inhibit Cdk2 activity *in vitro* using recombinant Cdk2/cyclinE and Cdk2/cyclinA complexes (data not shown)

### Cell death

**Figure 7:**
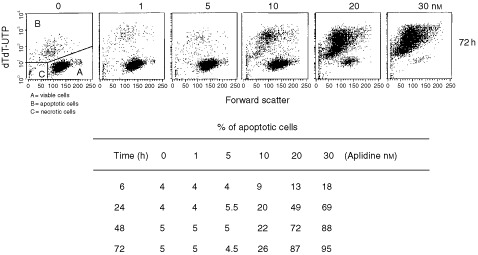
Detection of apoptosis. Cells were treated with different concentrations of Aplidine and the biparametric FSC/TdT-dUTP flow cytometric analysis were performed after 6, 24, 48 and 72 h after drug-washout. The percentage of apoptotic cells obtained at different time intervals and the flow cytometric FSC/TdT-dUTP analysis performed at 72 h after drug-washout are reported.

We investigated the mechanism of cell death induced by Aplidine by using TdT-dUTP flow cytometric analysis. One hour Aplidine exposure already induced apoptosis in Molt-4 cells at 6 h after drug-washout, and the fraction of apoptotic cells was dose and time dependent (Figure 7

). By using biparametric DNA/TdT-dUTP flow cytometric analysis we did not observe a specific cell cycle phase dependency of the apoptotic cell death (data not shown).

**Figure 8:**
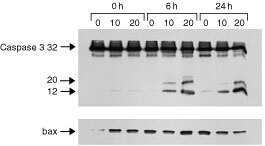
Western blot analysis of caspase 3 and Bax in Molt-4 cells treated for 1 h with 0, 10 or 20 nM Aplidine and evaluated at different time intervals after drug-washout.

To evaluate if caspase 3 was involved in the induction of apoptosis we performed Western blotting analysis on the total protein extract of Molt-4 cells treated for 1 h with 10 or 20 nM with Aplidine. The analysis was performed after drug treatment and at 6 and 24 h after drug-washout. As shown in Figure 8

we found that in the 10 nM treated cells just after 1 h treatment caspase 3 was cleaved into its 12 kDa activated form. At 6 and 24 h after drug-washout the activation of caspase 3 with the appearance of 12 and 20 kDa activated forms was clearly evident. Bax levels were not changed after treatment indicating that the apoptotic pathway was bax independent.

## DISCUSSION

The present study shows that Aplidine causes profound perturbations of the cell cycle as well as apoptosis in the human lymphoblastic leukaemia cell line Molt-4 at very low concentrations (nM range).

The concentrations (20 nM) which produced significant cell death can be achieved for several hours in the plasma of patients receiving Aplidine doses ranging from 3750 to 6000 μg m^−2^ given as a 24 h infusion (Paz-Ares *et al*, 2000; Armand *et al*, 2001).

A new finding, reported here for the first time is that Aplidine not only induces a G_1_ arrest (Crampton *et al*, 1984), but also a G_2_ blockade. It is interesting to note that the block in G_2_ is probably relevant as a large proportion of cells blocked in this phase subsequently undergo cell death. Whatever the mechanism of action of Aplidine, it acts very quickly and a prolonged exposure time (e.g. from 15 min to 1 or 24 h) does not increase the effects of the drug proportionally.

It has been proposed that Aplidine acts by inhibiting protein synthesis as suggested by the fact that the structural analogue didemnim inhibits the l α-elongation factor (Crews *et al*, 1994). Our data do not support the contention that protein synthesis is the primary mechanism by which Aplidine is cytotoxic. In fact, there are clear perturbations of the cell cycle at drug concentrations and time intervals in which no major decrease in the total synthesis of proteins or of other macromolecules occurs. For example, a concentration of 10 nM Aplidine for 1 h caused no detectable inhibition of total proteins, RNA or DNA synthesis but did cause marked cell cycle perturbations and cytotoxicity.

Another hypothesised mechanism of action of Aplidine was the inhibition of ODC (Urdiales *et al*, 1996). Intracellular polyamines, spermidine and spermine and their precursor putrescine are intimately involved in cell growth and proliferation. Intracellular polyamine levels are highly regulated and are primarily dependent upon the activity of ODC, that catalyses the first and rate-limiting step in polyamine biosynthesis. It has also been proposed that there exists polyamine-dependent restriction points during G_0_–G_1_ transition and G_1_ phase progression of the cell cycle (Harada and Morris, 1981; Seidenfeld *et al*, 1986; Charollais and Mester, 1988). The present study confirms that in cells treated with Aplidine there is a marked inhibition of ODC activity, an effect that could not be reproduced *in vitro* on the partially purified enzyme. The inhibition of ODC caused a strong decrease in putrescine levels which might be a relevant event triggering cell cycle perturbations and apoptosis. However, our data tend to exclude that depletion of putrescines plays a major role in the biological effects of Aplidine. In fact, the addition of putrescine at concentrations that increased intracellular putrescine concentrations comparable or even higher than that of controls did not significantly reduce cell cycle perturbations or the cytotoxicity of Aplidine.

Although further studies are required on this point it appears that the cell cycle perturbations observed after Aplidine treatment are not due to an effect on the cyclin dependent kinases that regulate the cell cycle progression. It appears more likely that the cell cycle block in G_1_ or in G_2_ is related to activation of cell cycle check points.

No information on the mechanism of cell cycle arrest is available but we can exclude the possibility that p53 is involved as Molt-4 cells do not express wt p53.

In addition, similar results have been obtained in other acute lymphoblastic leukaemia (ALL) lines with mutated p53 such as K562 and on fresh ALL cells grown on stromal feeder layer (Erba *et al*, in preparation).

In conclusion, although the data reported do not provide an explanation for the biological effects of Aplidine they indicate that this peptide acts by a mechanism different from that of other known anticancer drugs. The rapid induction of apoptosis in human leukaemia cells at concentrations even lower than those that can be achieved and maintained for many hours in plasma of patients receiving the drug at tolerable dose, provides a rational to undertake clinical investigation wit this drug in human leukaemia.

## References

[bib1] ArmandJPAdy-VagoNFaivreSChiezeSBaudinERibragVLecotFIglesiasLLopez-LazaroLGuzmanCJimenoJDucreuxMLe ChevalierTRaymondE2001 Phase I and pharmacokinetic study of aplidine (APL) given as a 24-hour continuous infusion every other week (q2w) in patients (pts) with solid tumor (ST) and lymphoma (NHL)[abstract]Proc Am Soc Clin OncolVol 20120aProceedings 37th ASCO Annual Meeting, May 12–15, San Francisco

[bib2] BergamaschiDRonzoniSTavernaSFarettaMDe FeudisPFairclothGJimenoJErbaED'IncalciM1999 Cell cycle perturbations and apoptosis induced by isohomohalichondrin B (IHB), a natural marine compound Br J Cancer 79 267277 9888468 10.1038/sj.bjc.6690044 PMC2362206

[bib3] BonfantiMTavernaSSalmonaMD'IncalciMBrogginiM1997 p21WAF1-derived peptides linked to an internalization peptide inhibit human cancer cell growth Cancer Res 57 14421446 9108443

[bib4] CharollaisRHMesterJ1988 Resumption of cell cycle in BALB/c-3T3 fibroblasts arrested by polyamine depletion: relation with “competence” gene expression J Cell Physiol 137 559564 3142887 10.1002/jcp.1041370323

[bib5] CramptonSLAdamsEGKuentzelSLLiLHBadinerGBhuyanBK1984 Biochemical and cellular effects of didemnins A and B Cancer Res 44 17961801 6713383

[bib6] CrewsCMCollinsJLLaneWSSnapperMLSchreiberSL1994 GTP-dependent binding of the antiproliferative agent didemnin to elongation factor 1alpha J Biol Chem 269 1541115414 8195179

[bib7] DepenbrockHPeterRFairclothGTManzanaresIJimenoJHanauskeAR1998 *In vitro* activity of aplidine, a new marine-derived anti-cancer compound, on freshly explanted clonogenic human tumour cells and haematopoietic precursor cells Br J Cancer 78 739744 9743292 10.1038/bjc.1998.570 PMC2062976

[bib8] DesiderioMA1992 Opposite responses of nuclear spermidine N8-acetyltransferase and histone acetyltransferase activities to regenerative stimuli in rat liver Hepatology 15 928933 1568734 10.1002/hep.1840150529

[bib9] DesiderioMAMatteiSBiondiGColomboMP1993 Cytosolic and nuclear spermidine acetyltransferases in growing NIH 3T3 fibroblasts stimulated with serum or polyamines: relationship to polyamine-biosynthetic decarboxylases and histone acetyltransferase Biochem J 293 475479 8343127 10.1042/bj2930475 PMC1134385

[bib10] ErbaEBergamaschiDBassanoLDamiaGRonzoniSFairclothGTD'IncalciM2001 Ecteinascidin-743 (ET-743), a natural marine compound, with a unique mechanism of action Eur J Cancer 37 97105 11165136 10.1016/s0959-8049(00)00357-9

[bib11] FairclothGPerezJFernandezJLSPAvilaJGarciaMErbaED'IncalciMCanedoAGarcia de QuesadaTJimenoJ1995 Marine depsipeptides with activity against solid tumour models[abstract]p529Proceedings 8th ECCO Congress, Paris, 29 October–2 November

[bib12] FairclothJGRinehartKNunez de CastroIJimenoJ1996 Dehydrodidemnin B a new marine derived antitumour agent with activity against experimental tumour models Ann Oncol 7 34

[bib13] GeldofAAMastbergenSCHenrarREFairclothGT1999 Cytotoxicity and neurocytotoxicity of new marine anticancer agents evaluated using *in vitro* assays Cancer Chemother Pharmacol 44 312318 10447579 10.1007/s002800050983

[bib14] HaradaJJMorrisDR1981 Cell cycle parameters of Chinese hamster ovary cells during exponential, polyamine-limited growth Mol Cell Biol 1 594599 9279373 10.1128/mcb.1.7.594-599.1981 PMC369707

[bib15] LoboCGarcia-PozoSGDe CastroINAlonsoFJ1997 Effect of dehydrodidemnin B on human colon carcinoma cell lines Anticancer Res 17 333336 9066673

[bib16] LowryOHRosenbroughNJFarrALRandallRJ1951 Protein measurement with folin phenol reagent J Biol Chem 193 265275 14907713

[bib17] MontalentiFSenaGCappellaPUbezioP1998 Simulating cancer-cell kinetics after drug treatment: application to cisplatin on ovarian carcinoma Phys Rev E 57 58775887

[bib18] Paz-AresLAnthonyAPronkLTwelvesCAlonsoSCortes-FunesHCelliNGomezCLopez-LazaroLGuzmanCJimenoJKayeS2000p86Proceedings 11th NCI-EORTC-AACR Symposium, November 7–10, Amsterdam

[bib19] SakaiRRinehartKLKishoreVKunduBFairclothGGloerJBCarneyJRNamikoshiMSunFHughesJrRGGravalosDGDe QuesadaTGWilsonGRHeidRM1996 Structure-activity relationships of the didemnins J Med Chem 39 28192834 8709112 10.1021/jm960048g

[bib20] SambrookJFritschEFManiatisT1989 Molecular Cloning: A Laboratory ManualCold Spring Harbor, NY: Cold Spring Harbor Laboratory Press

[bib21] SeidenfeldJBlockALKomarKANaujokasMF1986 Altered cell cycle phase distributions in cultured human carcinoma cells partially depleted of polyamines by treatment with difluoromethylornithine Cancer Res 46 4753 3079590

[bib22] SenaGOnadoCCappellaPMontalentiFUbezioP1999 Measuring the complexity of cell cycle arrest and killing of drugs: kinetics of phase-specific effects induced by taxol Cytometry 37 113124 10486523

[bib23] UbezioP1985 Microcomputer experience in analysis of flow cytometric DNA distributions Comput Programs Biomed 19 159166 3839734 10.1016/0010-468x(85)90007-8

[bib24] UchidaTWatanabeTKinoshitaTMurateTSaitoHHottaT1995 Mutational analysis of the CDKN2 (MTS1/p16ink4A) gene in primary B-cell lymphomas Blood 86 27242731 7670111

[bib25] UrdialesJLMorataPNunez de CastroISanchez-JimenezF1996 Antiproliferative effect of dehydrodidemnin B (DDB), a depsipeptide isolated from Mediterranean tunicates Cancer Lett 102 3137 8603376 10.1016/0304-3835(96)04151-1

